# Endoscopic ultrasound-guided cholecystoduodenostomy followed by stone clearance using electrohydraulic and mechanical lithotripsy in a frail patient with acute cholecystitis

**DOI:** 10.1055/a-2462-0975

**Published:** 2024-11-18

**Authors:** Francisco Vara-Luiz, Ivo Mendes, Gonçalo Nunes, Carolina Palma, Marta Patita, Jorge Fonseca, Pedro Pinto-Marques

**Affiliations:** 1 70816 Gastroenterology, Hospital Garcia de Orta EPE, Almada, Portugal; 2 112055 Aging Lab, Egas Moniz School of Health and Science, Caparica, Portugal

Laparoscopic cholecystectomy is considered the gold standard treatment of acute cholecystitis 1 ; however, endoscopic ultrasound-guided gallbladder drainage (EUS-GBD) is a valuable option for frail patients with high surgical risk 2 .

A 91-year-old dependent woman with several co-morbidities was admitted with acute pneumonia. During the period of hospitalization, she developed abdominal pain and fever. Abdominal computed tomography revealed gallbladder distension with one 20-mm stone and wall thickening, suggestive of acute cholecystitis. Given her advanced age, high surgical risk, and the evolving sepsis, cholecystectomy was avoided and EUS-GBD was performed ( Video 1 ).

Endoscopic ultrasound-guided cholecystoduodenostomy and subsequent stone clearance are performed in a frail elderly patient.Video 1

A linear echoendoscope was used, with the gallbladder being easily visualized from the duodenal bulb and common bile duct stones excluded. A 15 × 10-mm lumen-apposing metal stent (LAMS; Hot Axios) was successfully deployed creating a cholecystoduodenostomy ( Fig. 1 ), which led to clinical improvement, with resolution of the sepsis.

**Fig. 1:**
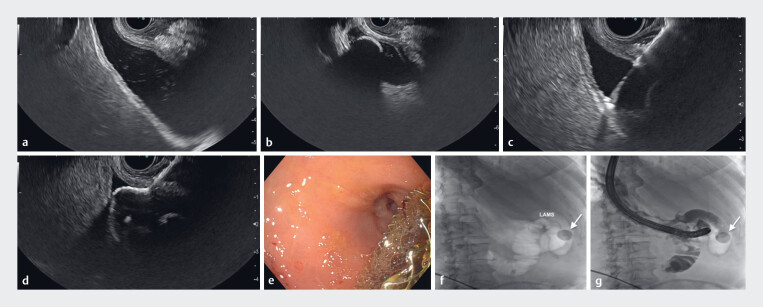
Images during endoscopic ultrasound (EUS)-guided cholecystoduodenostomy placement showing: **a** gallbladder thickening and distension, suggestive of acute cholecystitis; **b** a 20-mm gallstone; **c** puncture of the gallbladder with a 19G needle; **d** deployment of a 15-mm lumen-apposing metal stent (LAMS) under EUS view; **e** the cholecystoduodenostomy on endoscopic view; **f, g** fluoroscopic views of: **f** the LAMS; **g** the gallstone.

To assess the possibility of stone clearance and decrease the risk of further biliary events, an upper gastrointestinal endoscopy was performed 2 weeks later ( Fig. 2 ). A standard gastroscope was inserted through the LAMS to inspect the gallbladder, with mucosal congestion and one hard gallstone detected. Electrohydraulic lithotripsy (Autolith) was partially effective as it was impossible to achieve complete lumen filling and stone submersion with saline. Extraction of the gallstone fragments with a Dormia basket was attempted, but was complicated by stone retention and the need for a Soehendra lithotripter to remove the basket and complete fragmentation. The residual stones were then completely removed with a Rothnet retriever, and a 5-cm 10-Fr double-pigtail stent was placed to avoid long-term LAMS dysfunction. No procedural complications were observed and the patient remained asymptomatic during follow-up.

**Fig. 2:**
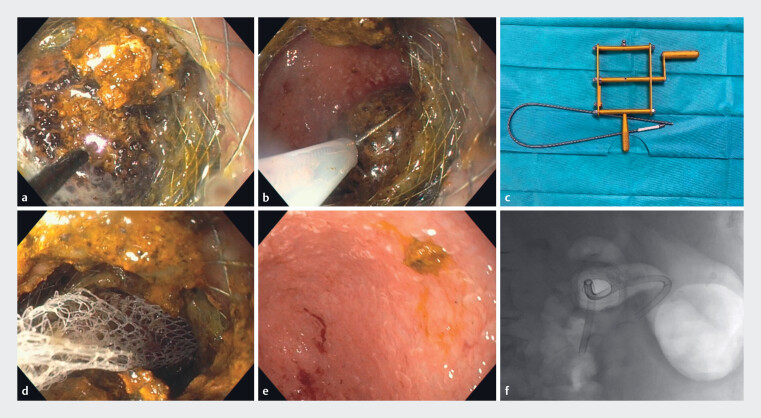
Images of the stone clearance procedure showing: **a** electrohydraulic lithotripsy being performed; **b** extraction of the gallstone fragments with a Dormia basket, which was complicated by stone retention; **c** a Soehendra lithotripter, which was used to remove the basket; **d** a Rothnet retriever being used to completely remove the residual stones; **e** the gallbladder mucosa with no evidence of residual stones; **f** the lumen-apposing metal stent and 5-cm 10-Fr double-pigtail stent on fluoroscopic view.

The advantages of EUS-GBD over a percutaneous approach include fewer adverse events and a lower reintervention rate, with similar technical and clinical success rates 3 4 . In patients who require prolonged biliary drainage, stone clearance should be considered, potentially reducing recurrence 3 4 . We demonstrate the effectiveness and safety of EUS-GBD in patients with acute cholecystitis who are unfit for surgery.

Endoscopy_UCTN_Code_TTT_1AS_2AH

## Notes

Endoscopy E-Videos https://eref.thieme.de/e-videos
*E-Videos* is an open access online section of the journal *Endoscopy* , reporting on interesting cases and new techniques in gastroenterological endoscopy. All papers include a high-quality video and are published with a Creative Commons CC-BY license. Endoscopy E-Videos qualify for HINARI discounts and waivers and eligibility is automatically checked during the submission process. We grant 100% waivers to articles whose corresponding authors are based in Group A countries and 50% waivers to those who are based in Group B countries as classified by Research4Life (see: https://www.research4life.org/access/eligibility/ ). This section has its own submission website at https://mc.manuscriptcentral.com/e-videos .
